# FastMRI Prostate: A public, biparametric MRI dataset to advance machine learning for prostate cancer imaging

**DOI:** 10.1038/s41597-024-03252-w

**Published:** 2024-04-20

**Authors:** Radhika Tibrewala, Tarun Dutt, Angela Tong, Luke Ginocchio, Riccardo Lattanzi, Mahesh B. Keerthivasan, Steven H. Baete, Sumit Chopra, Yvonne W. Lui, Daniel K. Sodickson, Hersh Chandarana, Patricia M. Johnson

**Affiliations:** 1 Center for Advanced Imaging Innovation and Research (CAI2R), Department of Radiology, New York University Grossman School of Medicine, New York, New York USA; 2 Bernard and Irene Schwartz Center for Biomedical Imaging, Department of Radiology, New York University Grossman School of Medicine, New York, New York USA; 3 Vilcek Institute of Graduate Biomedical Sciences, New York University Grossman School of Medicine, New York, New York USA; 4 Siemens Medical Solutions USA, New York, NY USA

**Keywords:** Databases, Cancer imaging

## Abstract

Magnetic resonance imaging (MRI) has experienced remarkable advancements in the integration of artificial intelligence (AI) for image acquisition and reconstruction. The availability of raw k-space data is crucial for training AI models in such tasks, but public MRI datasets are mostly restricted to DICOM images only. To address this limitation, the fastMRI initiative released brain and knee k-space datasets, which have since seen vigorous use. In May 2023, fastMRI was expanded to include biparametric (T2- and diffusion-weighted) prostate MRI data from a clinical population. Biparametric MRI plays a vital role in the diagnosis and management of prostate cancer. Advances in imaging methods, such as reconstructing under-sampled data from accelerated acquisitions, can improve cost-effectiveness and accessibility of prostate MRI. Raw k-space data, reconstructed images and slice, volume and exam level annotations for likelihood of prostate cancer are provided in this dataset for 47468 slices corresponding to 1560 volumes from 312 patients. This dataset facilitates AI and algorithm development for prostate image reconstruction, with the ultimate goal of enhancing prostate cancer diagnosis.

## Background & Summary

The introduction of the first fastMRI raw k-space datasets^1^ in 2020 sparked a surge of interest and transformative advancements in MR image reconstruction research. With the additional release of SKM-TEA^2^ in 2022, public raw k-space datasets have emerged as a catalyst for advancing MRI acquisition and reconstruction techniques, resulting in increased accelerations of imaging in both research and clinical settings. The availability of raw data not only facilitates familiar downstream AI processes such as classification and segmentation but also empowers upstream optimization of data acquisition and reconstruction. The impact of releasing raw k-space data is evident through quantifiable metrics including the large number of publications directly referencing fastMRI (~680), widespread participation in fastMRI-related public challenges^3,4^ and download statistics from Amazon Web Services (where fastMRI was ranked in 2022 as one of the top ten AWS-hosted life-science datasets). Furthermore, the introduction of subspecialist clinical annotations in fastMRI+^5^ in 2021 has enabled the development of clinically relevant reconstruction and classification frameworks, significantly enhancing the overall utility and effectiveness of the fastMRI dataset.

Prostate cancer (PCa) is a significant contributor to male cancer burden in the United States, accounting for 29% of diagnoses and a leading cause of cancer-related mortality^6^. The national economic impact of PCa is substantial, with annual healthcare costs exceeding $19 billion^7^. Projections indicate that approximately 34,700 deaths are expected to be attributed to PCa in 2023 in the United States^6^. Therefore, early detection strategies and improved management approaches are crucial for reducing mortality rates associated with PCa. MRI with T2-weighted and diffusion-weighted sequences is an effective diagnostic tool used in PCa management^8–11^. Accelerating imaging through advanced reconstruction techniques applied to under-sampled data holds the potential to enhance cost-effectiveness in prostate MRI workflows, while automated triage systems can contribute to increased efficiency and broader accessibility of prostate MRI. The PI-CAI dataset, introduced in 2022, consists of T2-weighted images and apparent diffusion coefficient maps. It underscored the importance of integrating AI into the prostate cancer (PCa) diagnostic pipeline. However, since it is limited to imaging data only, it also revealed an unmet need - the need for publicly available raw (k-space) prostate MRI data. To fill this gap, we launched the fastMRI prostate dataset aiming to contribute to the landscape of PCa MRI research. The fastMRI prostate dataset is a collection of raw T2-weighted and diffusion-weighted data procured from 3 T clinical systems. Crucially, this dataset incorporates slice, volume, and exam-level labels indicating the likelihood of clinically significant prostate cancer lesions. Thus, it not only enables the training of AI models for PCa diagnosis, but also opens avenues for optimizing image acquisition and reconstruction - a distinctive feature not present in previous datasets. In this paper, we describe the method for producing this dataset and demonstrate its potential uses for deep-learning-based reconstruction and automated diagnostics.

## Methods

**Fig. 1:**
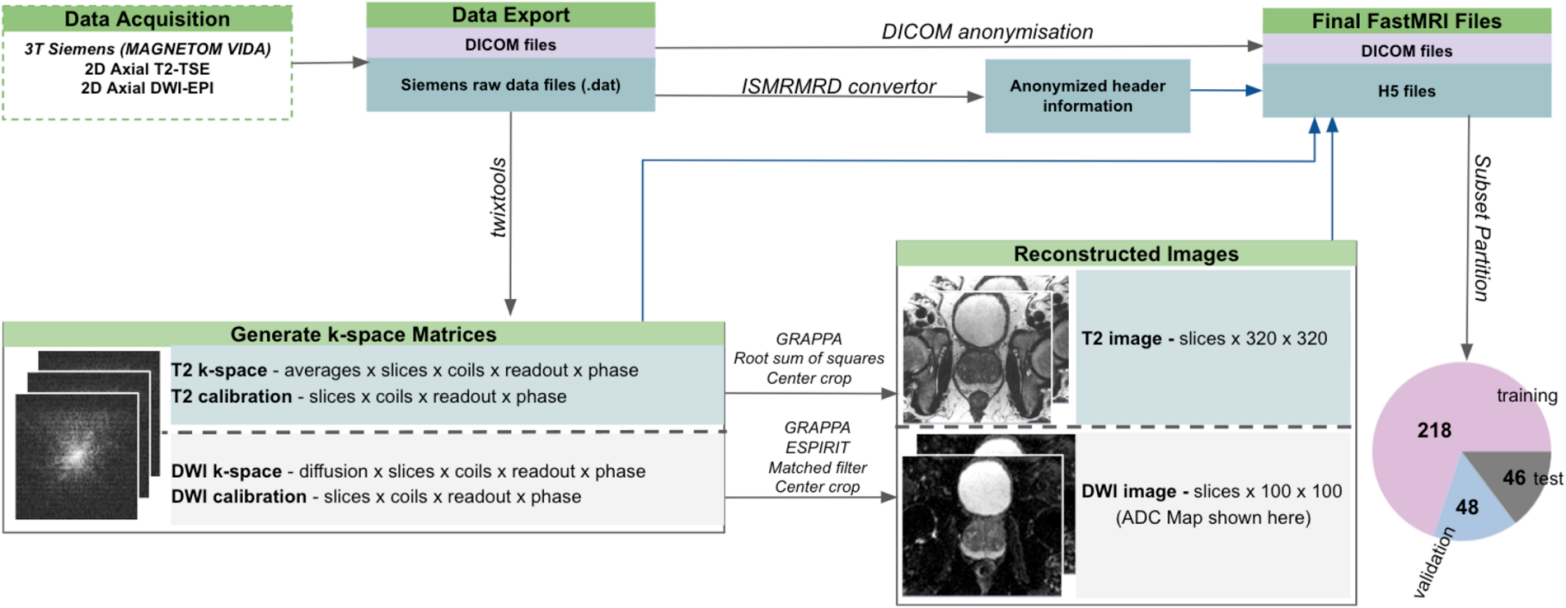
Generating the fastMRI prostate dataset. General workflow of data acquisition and processing used to generate the files in the fastMRI prostate dataset.

Figure 1 depicts the overall workflow utilized to generate the fastMRI prostate dataset. Biparametric 3 T MRI scans including T2-weighted and diffusion-weighted images (DWI) were acquired on a cohort of 312 patients. Raw data files along with the scanner-reconstructed DICOM files were exported from the scanner and anonymized. Offline reconstructions of the exported raw data were performed for T2-weighted and diffusion-weighted images. The reconstructed images are included along with the raw k-space data and DICOM images in the publicly released files. The exported DICOM files and the reconstructed images serve different purposes. The offline reconstructed images, together with the associated source code, function as a benchmark for developers creating their own image reconstruction algorithms. This allows for a direct comparison and validation of new methods against an established baseline, facilitating the development of potentially more advanced or specialized techniques. The DICOM files exported directly from the scanner encompass post-processing using proprietary scanner algorithms but are readily compatible with a range of pre-existing image processing and machine learning tools. The provision of DICOM files thereby ensures that our dataset can be seamlessly integrated into existing workflows and pipelines.

The number of image slices, image volumes and patients in both sequences for all data subsets are reported in Table 1.

**Table 1:** Number of image slices, image volumes and patients stratified by sequence and data subset type.

Data Subset	Sequence	Number of patients	Number of volumes	Number of slices
Train	T2W	218	218	6647
Train	DWI	218	872	26548
Validation	T2W	48	48	1462
Validation	DWI	48	192	5832
Test	T2W	46	46	1399
Test	DWI	46	184	5580

### Patient population

This dataset includes data from 312 male patients referred for clinical prostate MRI at NYU Langone Health between March 2020 and April 2021. The majority of this cohort (249/312) were referred for prostate MRI for pre-biopsy imaging following an elevated PSA test. For these patients with elevated PSA, the range of PSA values is 4–98 ng/mL with a mean of 8.79+/− 11.86 ng/mL. Other reasons for referral include active surveillance for patients with previously established risk of prostate cancer, post-treatment surveillance, and abnormalities on other exams (e.g pelvic CT or digital rectal exam). The mean age of the subjects was 66 ± 8 years. The collection and sharing of this dataset were approved by the NYU Langone Health Institutional Review Board (S18-00412, S23-00146) and were compliant with Health Insurance Portability and Accountability. A waiver of consent was approved by the institutional review board as the study uses anonymized data, and additionally, patient care was not impacted due to the retrospective nature of the study.

### Imaging protocol

Data were acquired on two clinical 3 T systems (MAGNETOM Vida, Siemens Healthineers, Germany), each with a matrix coil array made up of selected channels from an anterior body coil array and a posterior spine coil array (see Table 2 for range of number of coil elements selected). 2D axial T2-weighted turbo spin echo (TSE) and echo planar imaging (EPI)-DWI sequences were acquired for each patient. The T2-weighted data were acquired with 3 averages, the first and third averages sampling the odd lines of k-space, and the second average sampling the even lines of k-space. The EPI-DWI scans were performed using tri-directional diffusion-sensitizing gradients with *b-*values of 50 s/mm^2^ (B50) and 1000 s/mm^2^ (B1000), performed with 4 and 12 averages, respectively. The parallel imaging acceleration factor for EPI-DWI scans was R = 2. Additional scan parameters for both sequences are listed in Table 2.

**Table 2:** Scan parameters for the axial T2W TSE sequence and the EPI-DWI sequence.

Scan Parameter	T2W	DWI
TR (s)	3.5–7.2	5.0–7.3
TE (ms)	100	77
ETL	25	75
In-plane resolution (mm)	0.56 × 0.56*	2.0 × 2.0**
Slice thickness (mm)	3	3
Matrix size	320 × 320	100 × 100
FOV (mm)	180 × 180	200 × 200
Number of slices	30–36	24–38
Number of receive channels***	10–30	14–30

*these acquisitions have a phase resolution of 0.7, so the acquired data has a resolution of 0.56 × 0.80 mm which is then interpolated to 0.56 × 0.56 mm.

**the inline reconstructed DICOM images have been interpolated to a 1 mm × 1 mm in-plane resolution.

***combination of a body coil and a spine coil is used in acquiring this data, however, for a given scan, all coil elements may not be used depending on patient size and position, creating a variation in the number of receive channels.

### Raw data anonymization, processing and reconstruction

For both the T2 and diffusion sequences, the k-space data was exported directly from the scanner console while retaining all the averages and diffusion directions. The metadata was anonymized via conversion to the vendor neutral ISMRMRD format. The result was then stored as a dataset along with the k-space matrices in Hierarchical Data Format Version 5 (H5) format^12^. Emphasis was placed on retaining the same file format that was used for prior fastMRI datasets^1^, allowing existing codes (see Code Availability) for fastMRI to be run on this prostate dataset with minimal modification.

All python scripts to reconstruct the T2 and DWI images from the k-space data are included in the public GitHub repository: https://github.com/cai2r/fastMRI_prostate (see Code Availability). For the T2-weighted data, each under-sampled average is reconstructed using GeneRalized Autocalibrating Partially Parallel Acquisition (GRAPPA)^13^ with a root sum of squares coil combination. The three averages are then combined in image space. The DWI reconstruction includes EPI gridding, GRAPPA reconstruction, and an SNR-optimizing matched filter coil combination^14^ with coil sensitivities estimated using ESPIRIT^15^. The trace, ADC, and estimated B1500 are computed from the six individual source images. The reconstructed images were also stored in the H5 files as potential training targets for parallel imaging reconstruction. Code to load the reconstructed images from the H5 files and visualize them is provided in the GitHub repository as well.

**Fig. 2:**
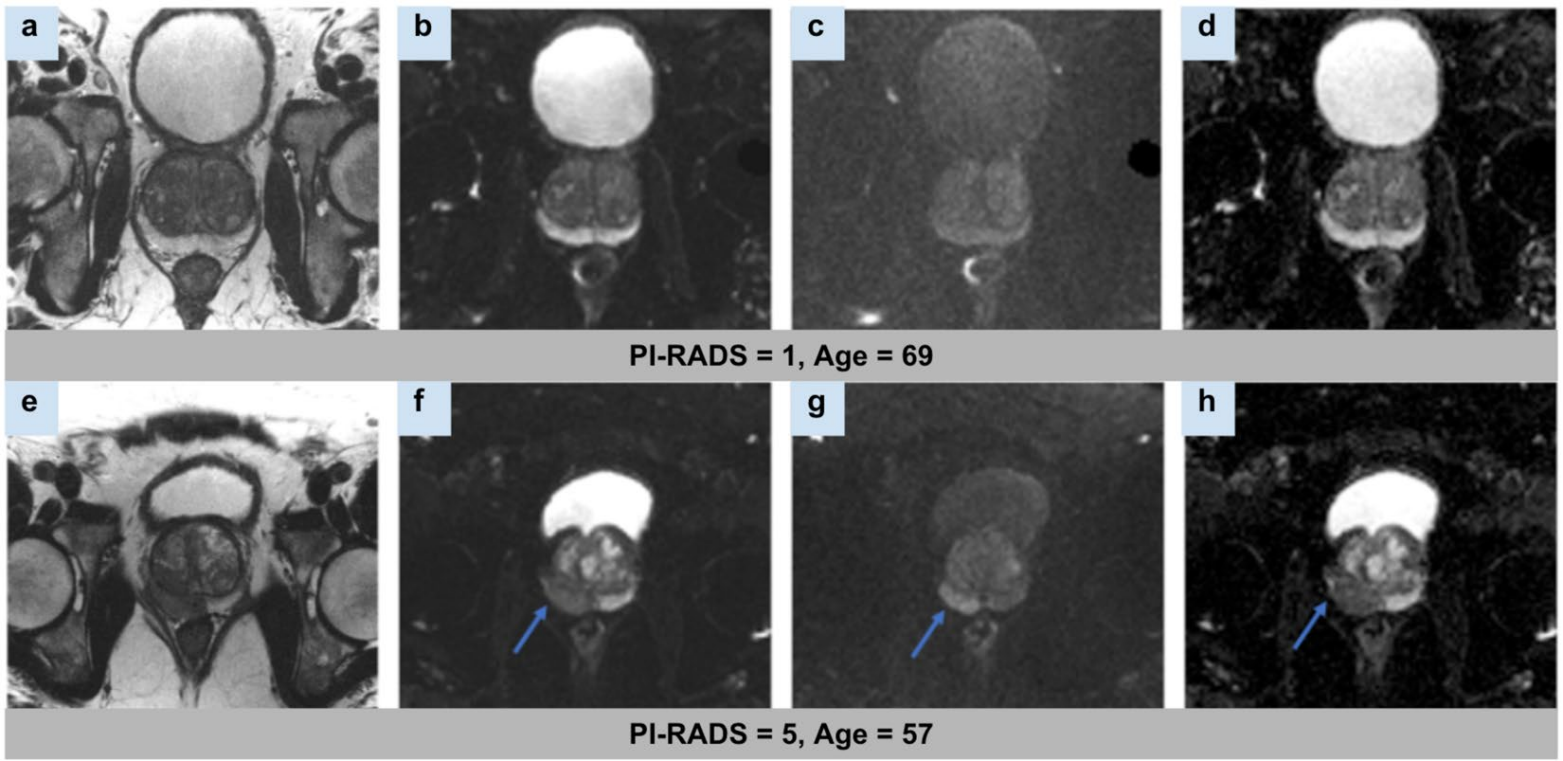
Example fastMRI prostate images. (Row 1) A reconstructed axial T2-weighted image (**a**), B50 DWI (**b**), B1000 DWI (**c**) and ADC Map (**d**) for a 69-year-old man referred for prostate evaluation, with a PI-RADS assessment of 1 based on the full set of acquired MR images. (Row 2) A reconstructed axial T2-image (**e**), B50 DWI (**f**) B1000 DWI (**g**) and ADC Map (**h**) for a 57-year-old man referred for clinical prostate MRI, with a PI-RADS assessment of 5. Blue arrows indicate a lesion in the right posteromedial midgland peripheral zone.

Figure 2 shows a single slice of the reconstructed T2-weighted image set, along with a reconstructed B50 trace, B1000 trace, and calculated ADC map for each of two subjects with PI-RADS scores 1 and 5, respectively.

### DICOM Data deidentification and processing

The scanner exported DICOM data for the T2-weighted and diffusion-weighted sequences were deidentified using a custom deidentification tool based on RSNA Clinical Trial Processing (CTP) Tool with additional custom modifications that have been recommended in the literature^16^. A thorough manual examination of each DICOM image to further screen for any potential instances of protected health information (PHI) was also conducted.

### Labels

A fellowship-trained radiologist reviewed all the T2 and DWI images, assigning a Prostate Imaging Reporting & Data System (PI-RADS v2.1) label to each slice of a patient volume on each sequence^17^. PI-RADS v2.1 assessment uses a 5-point scale based on the likelihood that findings correlate with the presence of a clinically significant cancer with the following categories: PI-RADS 1 – Very Low (highly unlikely it is clinically significant cancer), PI-RADS 2 - Low (unlikely it is clinically significant cancer), PI-RADS 3 – Intermediate (uncertain if it is clinically significant cancer), PI-RADS 4 – High (likely that it is clinically significant cancer), PI-RADS 5 – Very High (highly likely that it is clinically significant cancer). The images were labeled retrospectively for research purposes; the labels were not utilized to direct clinical care.

The T2 and DWI images were both labeled slice-by-slice in isolation. This is a deviation from how clinical interpretation of these images is performed, where a PI-RADS score would be assigned to each subject using information from the entire exam. For this dataset, we chose to provide slice-level labels because this results in a training dataset size on the order of the number of slices rather than the number of exams (with the caveat that nearby slices in the same patient will be correlated). Volume-level labels are derived as a maximum of the slice-level labels, and exam-level labels are derived as the combination of the T2- and diffusion-weighted sequences as per PI-RADS v2.1. Slice-, volume- and exam-level labels are provided in tabular files (csv format) in the Github repository and are also downloaded along with the data.

### Subset partition

**Fig. 3:**
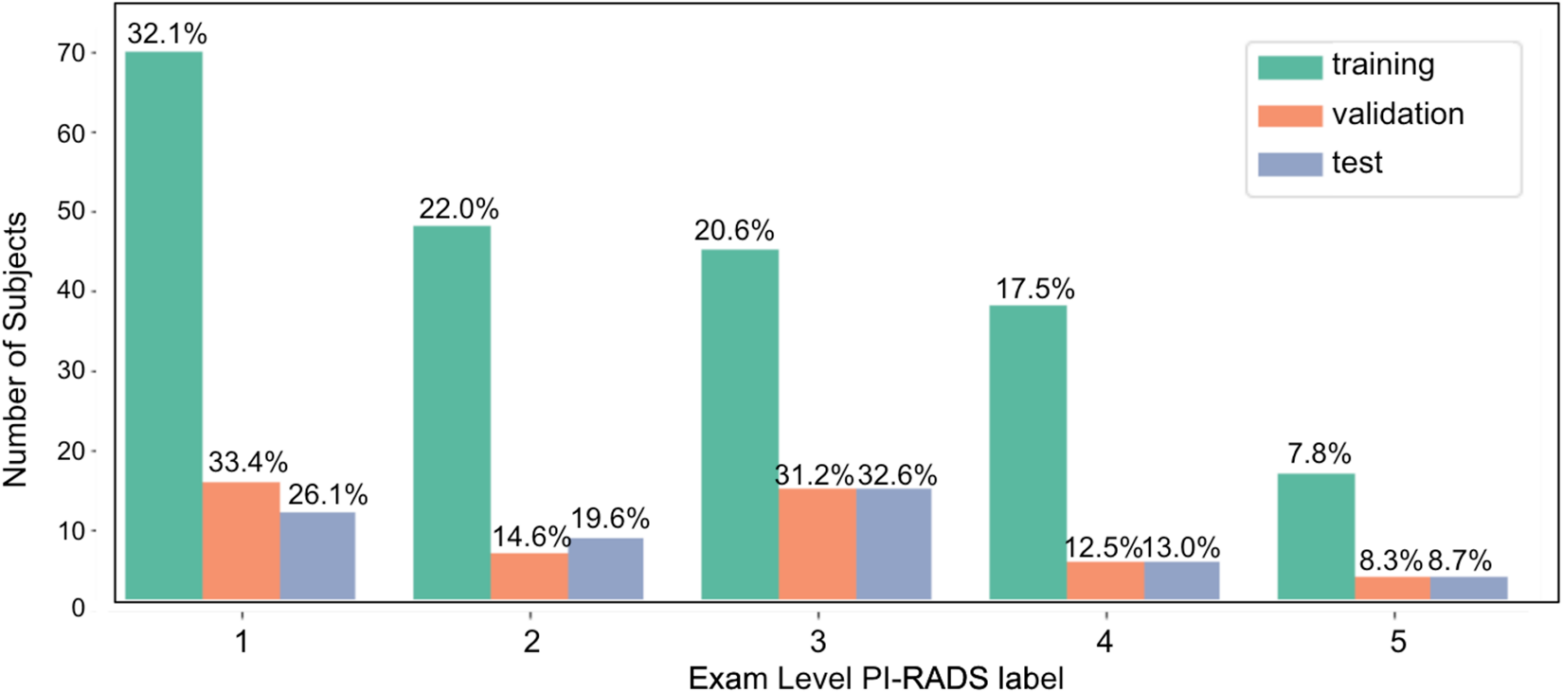
Number of exam level labels for each PI-RADS score in the training, validation and test subsets in the fastMRI prostate dataset. The percentage values indicate the distribution of PI-RADS scores within the training, validation and test subsets.

A suggested split of the cohort of 312 patients into training, validation, and test subsets, comprising 218, 48, and 46 subjects, respectively, is provided in the associated data (see Data Records). While the partitioning process was randomized, we verified that there is a representative distribution of exam-level PI-RADS scores within each subset. This approach aims to maintain a proportionate representation of samples during model training phases, fostering a comprehensive and unbiased analysis of the data. The exam level PI-RADS score distribution is seen in Fig. 3.

## Data Records

The H5 files and DICOM files for all 312 patients are available to download through the NYU catalog^18^. Training, validation and test folders are each provided separately for T2 and diffusion sequences for the ability to isolate and download a sequence of interest. The file naming convention for the H5 files is “file_prostate_sequence_fastmriID.h5” where the sequences can be “AXDIFF” or “AXT2” indicating axial diffusion and axial T2 respectively. The fastMRI ID ranges between 1 and 312, assigning a unique ID for each patient. The header fields included in each H5 file (diffusion and T2) are specified and described in Table 3. Field names were chosen to retain alignment with existing fastMRI file fields for easy use of the fastMRI code repository. For the DICOM files, each patient folder is labeled by its fastMRI ID (between 1–312) and consists of 4 sequence folders, described in Table 4. The slice, volume and exam-level PI-RADs label files are available for download from the NYU catalog^18^ and https://github.com/cai2r/fastMRI_prostate, in the data folder. Reconstruction scripts for both T2 and diffusion data are also available in the Github repository.

**Table 3:** Description of data fields of fastMRI prostate files.

Sequence	Field	Description
T2	ismrmrd_header	Anonymized metadata in the ISMRMRD standard data format
	kspace	Raw k-space data with dimensions (averages, slices, coils, readout, phase)
	reconstruction_rss	Reconstructed image volume with dimensions (slices, x, y)
	calibration_data	Calibration scan acquired with dimensions (slices, coils, readout, phase)
DWI	ismrmrd_header	Anonymized metadata in the ISMRMRD standard data formats
	kspace	Raw k-space data with dimensions (diffusion*, slices, coils, readout, phase)
	b50x, b50y, b50z	Reconstructed source image with *b* = 50 s/mm^2^ in the X, Y and Z directions with dimensions (slices, x, y)
	b1000x, b1000y, b1000z	Reconstructed source image with *b* = 1000 s/mm^2^ in the X, Y and Z directions with dimensions (slices, x, y)
	trace_b50, trace_b1000	Geometric mean of tri-directional source images with dimensions (slices, x, y)
	adc_map	Apparent diffusion coefficient map, calculated from the source images with dimensions (slices, x, y)
	calibration_data	Calibration scan acquired with dimensions (slices, coils, readout, phase)
	coil_sens_maps	Coil sensitivity maps calculated using ESPIRIT with dimensions (slices, coils, readout, phase)
	phase_correction	Phase correction scan acquired with dimensions (diffusion direction, slices, coils, readout, polarity**)
	b1500***	High b-value image with b = 1500 s/mm^2^ (calculated from source images) with dimensions (slices, x, y)

*the diffusion dimension (size 50) is comprised of 3 diffusion directions and 4 averages for the B50 images, 3 diffusion directions and 12 averages for the B1000 images, and 2 averages for B0 images.

**bipolar EPI readout trajectory.

***the calculated b1500 images are not used in the calculation of the ADC maps.

**Table 4:** DICOM folder descriptions within each patient folder.

Folder Name	Sequence Type
AX_DIFFUSION_ADC	Scanner calculated and exported ADC maps
AX_DIFFUSION_CALC_BVAL	Scanner calculated and exported B1500 DICOMS
AX_DIFFUSION_TRACEW	Scanner exported trace B50 and B1000 DICOMS
AX_T2	Scanner exported T2 DICOMS

## Technical Validation

Rather than showing new research outcomes, the demonstrations in this section are meant to be educational and indicative of data quality and robustness. The following experiments show that the dataset is applicable for deep learning reconstruction and classification tasks. The deep learning reconstruction experiments underscore the dataset’s image quality; specifically, the SSIM results are consistent with those from analogous studies on prostate MRI^19^ thereby confirming our dataset’s congruence with established clinical imaging standards and reinforcing its suitability for robust model training. The classifier’s performance attests to the diagnostic information contained within the images and the accuracy of the accompanying labels.

### Deep learning based reconstruction of accelerated prostate mri

An end-to-end variational network (VarNet) model^19,20^ was trained using the code from the fastMRI repository (https://github.com/facebookresearch/fastMRI) and the training subset of the publicly released fastMRI prostate data. Separate models were trained for reconstruction of each prostate imaging sequence type: axial T2-weighted and diffusion-weighted (B50 and B1000). For training the axial T2-weighted reconstruction model, image inputs were a single average with retrospective 4-fold under-sampling relative to Nyquist sampling, for an effective acceleration R = 6 relative to the clinical protocol (which used 3 averages and an under-sampling factor of 2). The target images for this model were the corresponding conventional reconstructions described in the Raw Data Anonymization, Processing and Reconstruction section.

For diffusion-weighted under-sampled reconstruction, the VarNet model was configured for joint reconstruction of all averages and diffusion directions. The diffusion data was acquired with twofold under-sampling. The image inputs were 1 average and 4 averages for the B50 and B1000, respectively, which resulted in ~3-fold acceleration overall, relative to the clinical protocol. Acceleration was achieved by reducing the number of averages, rather than further under-sampling k-space. The target images for this model were the conventional reconstructions of B50 and B1000 trace described in the Raw Data Anonymization, Processing and Reconstruction section. The VarNet model used here had an extra channel dimension to accommodate the multiple averages and diffusion directions. This extra dimension was retained throughout the network, and the averages were only combined at the final stage prior to calculating the loss at each iteration.

The networks were trained with an Adam optimizer, learning-rate = 1e-03 and batch size = 4. An SSIM- based loss function was used to train the network to minimize the distance between the predicted and target images and early stopping was used to prevent the network from overfitting.

**Fig. 4:**
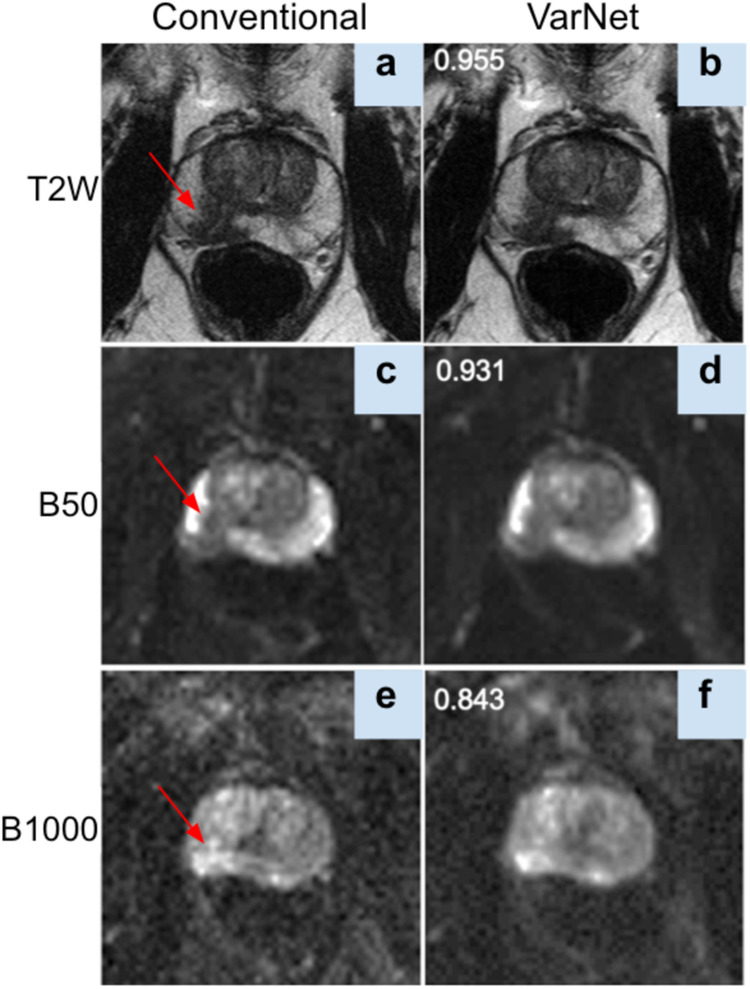
Representative example from the test set from the VarNet reconstruction with an effective acceleration R = 6 for T2-weighted (**a,b**) and R = 3 for the B50 (**c,d**) and B1000 (**e,f**) images. Red arrows indicate a lesion in the right posteromedial midgland peripheral zone, which is visible in both the conventional and accelerated reconstructed images. Numbers in images b-f correspond to SSIM values between the image and its conventional reconstruction counterpart.

The results demonstrate that the VarNet based reconstruction method produced high-quality images at high acceleration factors. The averaged SSIM computed for all image volumes in the test set was 0.952 ± 0.014, 0.930 ± 0.014 and 0.855 ± 0.061 for T2, B50 trace and B1000 trace respectively. A representative example from the test set comparing the conventional reconstruction to the VarNet reconstruction is shown in Fig. 4.

### Deep learning based classification of clinically significant prostate cancer

A 2D classification model was trained to diagnose clinically significant prostate cancer (csPCa) using the training subset of the publicly released fastMRI prostate data. The ConvNext architecture^21^, initialized with ImageNet weights, was selected for this task, and two distinct models were trained: one utilizing reconstructed T2-weighted image slices and the other employing calculated B1500 and ADC maps from the released H5 files. The ConvNext architecture was modified to accommodate 1 channel for the T2 images and 2 channels for the diffusion models instead of the default 3 channels. The models were trained on the provided training subset (on a slice-level basis, see Table 1; “number of slices” for exact subset splits). The released labels (PI-RADS scores) were used for training these networks. For a binary classification, the labels were binarized with a threshold of 2, where PI-RADS > 2 indicates csPCa requiring biopsy or other follow-up, and PI-RADS ≤ 2 indicates low risk for csPCa. Prior to network input, the T2-weighted and B1500 images were normalized by subtracting the mean value and then dividing by the standard deviation of the image. The ADC maps were normalized by applying a maximum threshold calculated as the 99th percentile of all the ADC maps in the training data, followed by scaling between 0 and 1 using the maximum value within each image. To prevent overfitting, early stopping and image augmentation techniques were employed, including translation, rotation, and flipping (in the left-right direction), for both networks. For the images in the T2 network, intensity rescaling was performed by clipping the intensities within each slice to within their 2^nd^ and 98^th^ percentile values as an additional augmentation technique. The T2 images were center cropped to a size of 224 × 224, while the diffusion images were resized to match this dimension using a bicubic interpolation.

For both networks, a binary cross entropy function was used as the loss function with an AdamW optimizer^22^, learning-rate = 0.001, weight decay = 1e-04 and batch size = 32. To address class imbalance, the cross-entropy function was weighted based on the number of positive cases in the training subset.

**Fig. 5:**
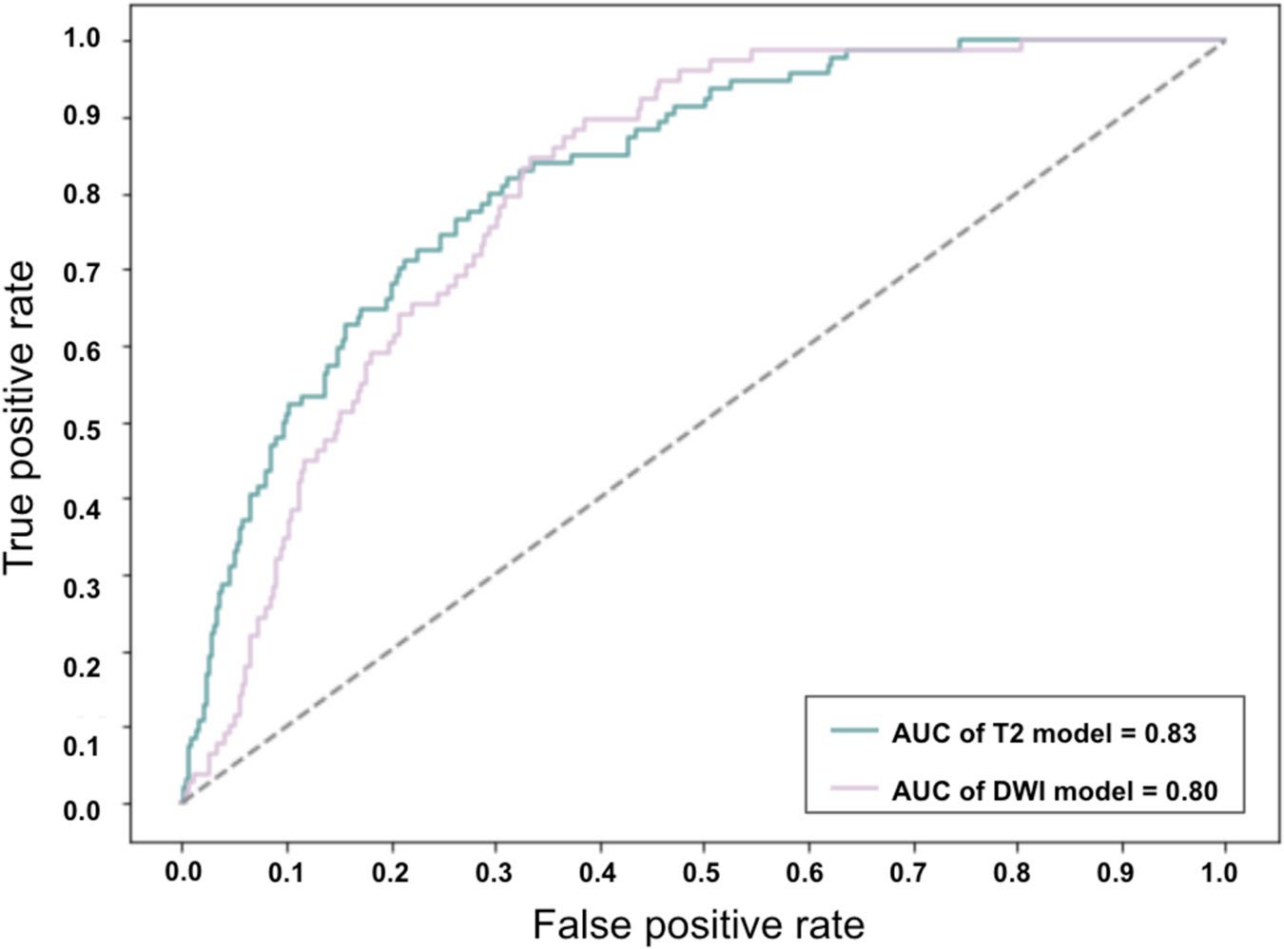
Receiver Operating Characteristic curves showing the diagnostic performance of the T2 and diffusion classification models on the test set (based on slice-level labels). The area under the ROC curve (AUC) achieved for the T2 test images was 0.83 (95% confidence interval: 0.77–0.87), while the AUC for the diffusion test images was 0.80 (95% confidence interval: 0.74–0.86).

The results on the released test set (same as the one used in the VarNet) demonstrate the feasibility of diagnosing csPCa from the provided data subsets using slice-level labels. In identifying csPCa in the T2 test images, an area under the receiver operating characteristic curve (AUC) of 0.83 (95% confidence interval: 0.77–0.87) was achieved. Similarly, for the diffusion test images, an AUC of 0.80 (95% confidence interval: 0.74–0.86) was obtained. Figure 5 displays the ROC curves of both models applied to the test subset.

## Usage Notes

The fastMRI prostate dataset provides an opportunity for the development of data-driven approaches in image reconstruction. The technical validation studies conducted have demonstrated the suitability of our multi-contrast data for investigating accelerated imaging methods. As is the case for the fastMRI + initiative, the inclusion of labels in this dataset will facilitate task-based evaluation of image reconstructions and joint training of reconstruction and classification models. The overarching objective is to enhance the efficacy of MRI in the detection and evaluation of prostate cancer. The dataset also increases the diversity of raw data available for the development of machine-learning algorithms tailored to medical imaging.

Although the fastMRI prostate dataset is smaller than the fastMRI brain and knee datasets, its significance lies in the utilization of real-world clinical data obtained from patients referred to our institution for suspected or known prostate cancer. Consequently, this dataset enables the development of clinically relevant AI models that have the potential to positively impact clinical care. Additionally, it is worth noting that, to our knowledge, the fastMRI prostate dataset represents the first public release of raw k-space data for an MR diffusion sequence.

It is important to note that, in certain cases, the offline reconstructions provided in the dataset exhibit inferior image quality when compared to the inline reconstructions that utilize proprietary algorithms on commercial scanners. We encourage the scientific community to explore avenues for improving both the image quality and computational efficiency of these offline reconstructions. As is ubiquitous in clinical practice, the diffusion sequence for fastMRI prostate is acquired with an acceleration factor R = 2 and is therefore not a fully sampled dataset. A single reader retrospectively labeled these images for research purposes. For future studies, we plan on including multiple readers to report inter-reader variability values. Furthermore, it should be emphasized that our trained models are not intended to be state-of-the-art, but are rather meant to serve as demonstrations of the technical characteristics associated with this dataset and its corresponding labels. The release of this data to the wider community is intended to foster the development of state-of-the-art deep learning models and reconstruction techniques that have clinical relevance and impact.

The fastMRI prostate dataset^18^ is hosted under a data usage agreement which may be found at https://fastmri.med.nyu.edu/.

## Data Availability

Code to load, reconstruct, and visualize fastMRI prostate data, as well as code to run training, validation, and testing for the deep-learning-based classification and reconstruction, may be found at https://github.com/cai2r/fastMRI_prostate. The fastMRI repository (which also contains code for the VarNet) may be found at https://github.com/facebookresearch/fastMRI.
